# The Role of [^68^Ga]Ga-Pentixafor PET/CT or PET/MRI in Lymphoma: A Systematic Review

**DOI:** 10.3390/cancers14153814

**Published:** 2022-08-05

**Authors:** Domenico Albano, Francesco Dondi, Francesco Bertagna, Giorgio Treglia

**Affiliations:** 1 Nuclear Medicine Department, ASST Spedali Civili Brescia, 25126 Brescia, Italy; 2 Department of Medical and Surgical Specialties, Radiological Sciences, and Public Health, Nuclear Medicine, University of Brescia, 25121 Brescia, Italy; 3 Clinic of Nuclear Medicine, Imaging Institute of Southern Switzerland, Ente Ospedaliero Cantonale, 6500 Bellinzona, Switzerland; 4 Department of Nuclear Medicine and Molecular Imaging, Lausanne University Hospital and University of Lausanne, 1015 Lausanne, Switzerland; 5 Faculty of Biomedical Sciences, Università della Svizzera Italiana, 6900 Lugano, Switzerland

**Keywords:** PET/CT, PET/MRI, Pentixafor, CXCR4, gallium-68, lymphoma, nuclear medicine

## Abstract

**Simple Summary:**

Preliminary evidence about the useful impact of [^68^Ga]Ga-Pentixafor PET/CT or PET/MRI in lymphoma is available but reveals heterogeneous findings. The aim of this systematic review was to analyze the published data about the role of [^68^Ga]Ga-Pentixafor PET/CT or PET/MRI in lymphoma and to focus on different lymphoma variants and clinical fields. [^68^Ga]Ga-Pentixafor PET may be considered a useful imaging method for staging and treatment response evaluation of several lymphomas, mainly FDG-not-avid variants. These findings may change the diagnostic flow-chart of lymphoma in the future.

**Abstract:**

The aim of this systematic review was to investigate published data about the role of gallium-68 Pentixafor positron emission tomography/computed tomography ([^68^Ga]Ga-Pentixafor PET/CT) or PET/magnetic resonance imaging (PET/MRI) in patients affected by lymphoma. A comprehensive computer literature search of the Scopus, PubMed/MEDLINE, and Embase databases was conducted including articles indexed up to June 2022. In total, 14 studies or subsets in studies were eligible for inclusion. From the analyses of the selected studies, the following main findings have been found: (1) lymphomas can be considered [^68^Ga]Ga-Pentixafor avid diseases, also in cases of fluorine-18 fluorodeoxyglucose [^18^F]FDG-not avid forms such as lymphoplasmacytic lymphoma (LPL), chronic lymphocytic leukemia (CLL), marginal zone lymphoma (MZL) and central nervous system lymphoma (CNSL); (2) among lymphomas, mantle cell lymphoma (MCL) and MZL are those with highest [^68^Ga]Ga-Pentixafor uptake; (3) [^68^Ga]Ga-Pentixafor PET/CT or PET/MRI is a useful tool for the staging and treatment response evaluation; (4) [^68^Ga]Ga-Pentixafor PET seems to have a better diagnostic performance than [^18^F]FDG PET in evaluating lymphomas. Despite several limitations affecting this analysis, especially related to the heterogeneity of the included studies, [^68^Ga]Ga-Pentixafor PET may be considered a useful imaging method for staging and treatment response evaluation of several lymphomas, especially MZL, CNSL and LPL.

## 1. Introduction

The complex chemokine network, which consists of a super-family of small structurally related cytokines [1], influences the growth, the migration and the survival of several cell types, even tumoral cells. These chemokines and their receptors may be expressed by the tumor cells and the stromal components [2]. The C-X-C motif chemokine receptor 4 (CXCR4) has been established as a potential target for various applications in oncology and interacts with crucial factors for cancer spread, such as angiogenesis or further involvement leading to therapeutic resistance [3]. CXCR4 is expressed in hematopoietic stem and progenitor cells in the bone marrow and by T and B lymphocytes, monocytes, macrophages, neutrophils, and eosinophils. Moreover, it is overexpressed on tumor cell surfaces in a large variety of solid and hematological cancers, including different lymphoma, multiple myeloma (MM), and chronic lymphocytic leukemia (CLL) [4,5]. For these reasons, the G-protein coupled receptor may be considered an ideal target for imaging and treatment of lymphoproliferative disorders. Consequently, radiotracers targeting CXCR4 for single-photon emission computed tomography (SPECT) and positron emission tomography (PET) have been developed [6,7,8]. [^68^Ga]Ga-Pentixafor is the best-known CXCR4 radiopharmaceutical used with promising results in various hematological and solid malignancies (i.e., lung cancer, pancreatic cancer, melanoma, breast cancer, liver cancer, and gliobastoma). Concerning imaging of lymphoproliferative diseases, fluorine-18 fluorodeoxyglucose [^18^F]FDG PET/computed tomography (PET/CT) or PET/magnetic resonance imaging (PET/MRI) are the most used hybrid imaging methods in staging, restaging and treatment response assessment [9]. The role of [^68^Ga]Ga-Pentixafor PET in lymphoproliferative disorders is not well defined. Moreover, according to a theranostic approach combining imaging and therapy, CXCR4-targeted therapy using Lutetium-177/Yttrium-90 [177Lu]Lu/[90Y]Y-PentixaTher has been also proposed [10,11] despite the limited experience on this application [12]. Before CXCR4-targeted therapy, a diagnostic confirmation of [^68^Ga]Ga-Pentixafor uptake is mandatory to confirm tumoral CXCR4 expression and maximize the efficacy of radioactive treatment [13]. The aim of this systematic review was to investigate the published data about the role of [^68^Ga]Ga-Pentixafor PET in lymphoma to better understand the role of this imaging technique as an alternative or complementary method compared to [^18^F]FDG PET/CT or PET/MRI.

## 2. Materials and Methods

The systematic review was conducted according to the PRISMA statement [14] and the review question was to investigate the diagnostic role of [^68^Ga]Ga-Pentixafor PET in patients with lymphoma.

### 2.1. Search Strategy

Taking into account the review question, a comprehensive literature search of the Scopus, PubMed/MEDLINE, and Embase databases was conducted to find relevant published articles about the role of [^68^Ga]Ga-Pentixafor PET/CT or PET/MRI in patients affected by lymphoma. We used a search algorithm based on a combination of the following words: (a) “PET” OR “PET/CT” OR “PET/MRI” OR “positron emission tomography” AND (b) “lymphoma” OR “lymphoproliferative” AND (c) “CXCR4” or “Pentixafor”. No limitation regarding the study period was applied, and the search was updated until 30 June 2022. Only articles in the English language were selected. To enlarge our research, the references of the retrieved articles were also screened for searching for additional papers. All reports collected were managed using EndNote^®^Basic (ThompsonReuters).

### 2.2. Study Selection

Studies or subsets in studies investigating the diagnostic role of [^68^Ga]Ga-Pentixafor PET in patients with lymphoma were eligible for inclusion. Instead, exclusion criteria were: (a) articles not in the field of interest; (b) meta-analyses, reviews, letters, conference proceedings, editorials, abstracts in the field of interest; and (c) case reports or small case series (less than 5 patients included) in the field of interest. Two researchers (GT and DA) independently reviewed the titles and abstracts of the records, applying the above-mentioned inclusion and exclusion criteria and the same two researchers then independently reviewed the full-text version of the articles to evaluate their suitability. In case of disagreement, a third opinion (FB) was asked.

### 2.3. Quality Assessment

The quality assessment included assessment of both the risk of bias and applicability concerns using QUADAS-2 evaluation [15].

### 2.4. Data Abstraction

For each included study, data were collected concerning the basic study features (first author name, year of publication, country, study design), the main clinical patients features (age, gender), technical variables (PET device used, radiotracer injected activity, uptake time, image analysis), number of patients evaluated and type of lymphomas. The main findings of the papers analyzed in this review are described in the “Results” section.

### 2.5. Statistical Analysis

Due to the heterogeneity of the target condition (different types of lymphomas), we have planned a systematic review (qualitative synthesis) without meta-analysis (quantitative synthesis). Therefore, a statistical analysis (pooled analysis) was not performed.

## 3. Results

### 3.1. Literature Search

**Figure 1:**
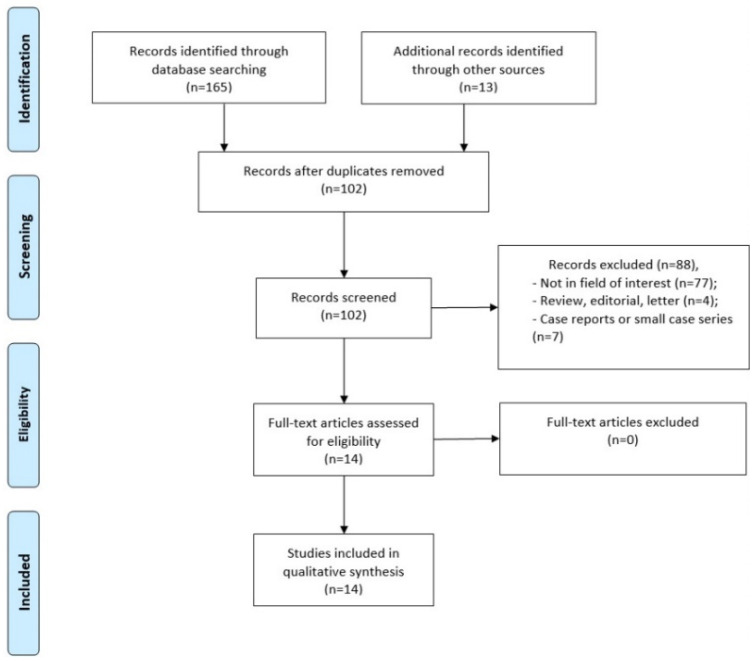
PRISMA literature search flow-chart.

**Figure 2:**
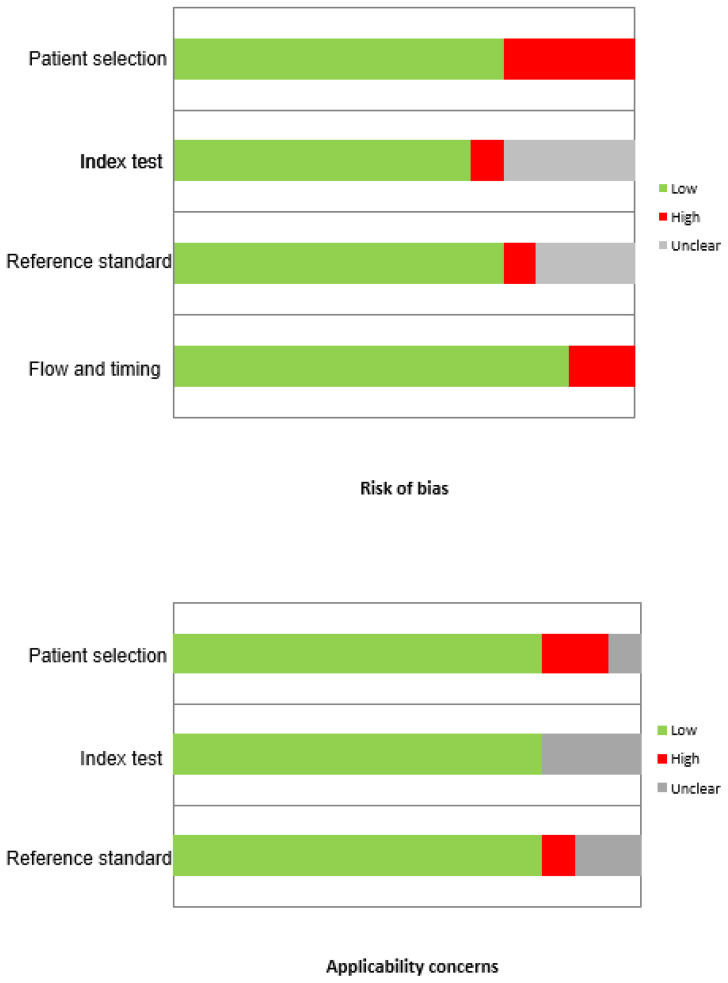
Quadas 2 score of the studies included in the review.

The comprehensive computer literature search from the Scopus, PubMed/MEDLINE and Embase databases revealed 102 studies collected and managed by EndNote^®^Basic (ThompsonReuters, Toronto, ON, Canada). Reviewing titles and abstracts, 77 studies were excluded since the reported data were not within the field of interest of this review; 11 articles were excluded as case reports, small case series, editorials, comments, reviews or conference proceedings. Lastly, 14 articles were selected for this systematic review [15,16,17,18,19,20,21,22,23,24,25,26,27,28]. No additional studies were found viewing the references of these articles (Figure 1). Results of the quality assessment of included studies is reported in Figure 2. In the majority of the studies the risk of bias or applicability concerns was low. Four studies had a high risk of bias for patient selection due to the unexplained patient exclusions. Only one study had high risk of bias for the index test, having the observers not blinded to clinical contest and patient’s anamnesis. Reference standard was not explained in three articles. The main features of the included studies are summarized in Table 1 and Table 2.

### 3.2. Qualitative Analysis

Among 14 articles that included 472 patients affected by lymphoma [16,17,18,19,20,21,22,23,24,25,26,27,28,29], 6 were of retrospective nature [19,20,22,23,27,28] and 7 were prospective studies [16,17,18,21,24,25,26,29]. In most studies, hybrid PET/CT was used [17,19,22,23,26,27,28], with the exception of six articles [16,18,21,24,25,29] where only PET/MRI was used and one where both PET/CT and PET/MRI scanners were applied [20]. PET/CT images were analyzed visually and semi-quantitatively in all cases. PET was considered positive in presence of lesion with radiotracer uptake higher than the surrounding background and blood pool activity, excluding sites of physiological or inflammatory uptake. Maximum standardized uptake value (SUVmax) was the most frequent semiquantitative measure used, followed by SUVmean and SUV ratios (ratio of SUV in the lesion and in the background, with liver and blood pool as background). No studies investigated texture analysis. The activity injected was wide ranging from 84 to 185 MBq according to each institutional protocol. Instead, uptake time (from radiopharmaceutical injection to image acquisition) was in almost all cases 60 min.

### 3.3. Lymphoma Variants Analysis

The most common histological type of lymphoma investigated in the included studies was marginal zone lymphoma (MZL) with a total of 276 patients recruited, followed by central nervous system lymphoma (CNSL) with 54 cases, mantle cell lymphoma (MCL) with 46 cases, Waldenstrom macroglobulinemia/lymphoplasmacytic lymphoma (WM/LPL) with 40 cases, chronic lymphocytic leukemia (CLL) with 24 cases and T-cell lymphoma with 11 cases, and others such follicular lymphoma (FL), diffuse large B cell lymphoma (DLBCL), or not specified in the remaining 19 patients. Among mucosa-associated lymphatic tissue (MALT) lymphoma was the most frequent variant with 78 cases.

### 3.4. MZL/MALT

[^68^Ga]Ga-Pentixafor PET demonstrated a high detection rate in detecting MZL, both with PET/CT and PET/MRI devices. Haug et al. [18] demonstrated increased uptake in 33/36 patients affected by MALT lymphoma (10 gastric, 11 orbital, 5 pulmonary, 3 soft-tissues; 1 adrenal gland, 1 tonsillary, 1 parotid gland, and 1 urinary bladder), with only 3 cases of negative PET but in patients with orbital MALT after surgical removal of the lesion. In all positive [^68^Ga]Ga-Pentixafor PET/CT, the uptake was very high with a mean SUVmax of 8.6 ± 4.7, a mean SUVmean of 4.7 ± 1.8, and mean SUVpeak of 8.0 ± 4.2. Also Duell et al. [22] obtained an excellent detection rate of [^68^Ga]Ga-Pentixafor PET/CT in 22 MZL (15 MALT lymphomas) with 20 true positive and 2 true negative scans, and with a diagnostic performance even better than conventional studies ([^18^F]FDG PET/CT, endoscopy and bone marrow biopsy). The radiopharmaceutical uptake in these patients was higher than those reported by Haug et al. [18] with a mean SUVmax of 13 ± 6.4. The high avidity of [^68^Ga]Ga-Pentixafor was confirmed by a recent study on a large sample of patients (n = 187 MZL), where, in comparison with other neoplastic diseases, this lymphoma variant is one of the most [^68^Ga]Ga-Pentixafor-avid after MM, adrenocortical carcinoma and adenoma, MCL and small cell lung cancer [27]. Another role of this radiotracer in MZL is related to the diagnostic properties of [^68^Ga]Ga-Pentixafor PET affecting the stage and the subsequent management; [^68^Ga]Ga-Pentixafor PET/CT significantly impacted staging results in almost half of patients (upstaging in seven cases and downstaging in three cases) and subsequently changed the treatment protocols in one-third of cases (n = 8) [22]. Considering treatment response evaluation, only one article is available [29] after Helicobacter Pylori eradication. Twenty-six patients affected by gastric MALT and twenty control patients without lymphoma underwent [^68^Ga]Ga-Pentixafor PET/MRI after Helicobacter Pylori eradication and PET findings were compared with biopsies to find complete remission. [^68^Ga]Ga-Pentixafor PET/MRI showed to be an excellent tool with pooled accuracy, sensitivity, specificity, positive and negative predictive values for the detection of residual gastric disease of 97%, 95%, 100%, 100%, and 92.9%, respectively. All gastric lymphomas were positive at PET/MRI with a mean SUVmax of 9.1 ± 0.7, a mean SUVmean of 4.5 ± 0.3, and SUV values were significantly higher compared to control patients.

### 3.5. CNSL

First, Herhaus et al. [20] studied 11 CNSL (8 primary and 3 secondary CNSL) with [^68^Ga]Ga-Pentixafor PET/CT or PET/MRI. In cases of active disease, [^68^Ga]-Pentixafor PET showed excellent contrast with the surrounding brain parenchyma. Ten patients had positive PET showing the presence of increased uptake corresponding to the brain lesion. The only patient who showed no tracer uptake had undergone extensive neurosurgical biopsy of the lymphoma and four weeks of steroid therapy before PET. In this patient, an MRI scan was negative. Moreover, in this study [20] the authors demonstrated that the pre-treatment PET uptake was correlated with treatment response evaluated by MRI. Particularly, CNSLs with low CXCR4 expression at initial PET were associated with better survival. Similar evidences were derived by Starzer et al. [25] in 7 patients (5 primary and 2 secondary CNSL) who performed 12 [^68^Ga]Ga-Pentixafor PET/MRI studies (7 for staging and 5 for follow-up purposes). The accuracy of PET in staging was perfect (100%) with 18 lesions detected with increased uptake and all confirmed by MRI scans. Also in the post-treatment field, PET/MRI results were in agreement with MRI findings. Chen et al. [28] compared [^68^Ga]Ga-Pentixafor PET/CT performance with [^18^F]FDG PET/CT in 26 patients. The detection rate of these two examinations was very similar: in a patient-based analysis, the findings with [^68^Ga]Ga-Pentixafor PET/CT were the same as those obtained by [^18^F]FDG PET/CT in all cases, except one where five further lesions were recognized by [^68^Ga]Ga-Pentixafor scan. However, [^68^Ga]Ga-Pentixafor PET/CT showed a remarkably higher tumor-to-background ratio compared to [^18^F]FDG PET/CT (21.93 ± 10.77 vs. 4.29 ± 2.16, *p* < 0.001).

### 3.6. WM/LPL

Three studies [17,19,26] on WM/LPL from the same group were available. In the first study [17], [^68^Ga]Ga-Pentixafor PET/CT diagnostic accuracy in studying WM/LPL was compared to [^18^F]FDG PET/CT. [^68^Ga]Ga-Pentixafor PET/CT showed a better diagnostic performance considering a global patient-based analysis (100% vs. 58.8%, *p* = 0.023), or sub-analysis for the evaluation of bone marrow disease (94.1% vs. 58.8%, *p* = 0.077), and lymph nodal involvement (76.5% vs. 11.8%, *p* = 0.003). In contrast, in the evaluation of paramedullary and CNS disease, [^68^Ga]Ga-Pentixafor PET/CT recognized more lesions than [^18^F]FDG PET/CT, despite no statistically significant differences. Also in the treatment response setting [26], [^68^Ga]Ga-Pentixafor PET/CT showed excellent performance after chemotherapy with a perfect agreement with clinical response classification [30] in 13/15 patients. The only two with different evidence were classified with very good partial response according to Owen et al. [30] and at complete response with [^68^Ga]Pentixafor. Instead, [^18^F]FDG PET/CT showed an agreement with clinical response in only one-third of cases (n = 5). Pan et al. [19] confirmed the higher detection rate of [^68^Ga]Ga-Pentixafor PET/CT compared to [^18^F]FDG PET/CT studying different lymphoma variants (DLBCL, FL, MZL, LPL). Among eight LPL patients, [^68^Ga]Ga-Pentixafor PET/CT was positive in all cases, while [^18^F]FDG PET/CT in five patients only. Moreover, in comparison to [^18^F]FDG PET, [^68^Ga]Ga-Pentixafor PET demonstrated more extensive disease and higher uptake in LPL and MZL than other lymphomas. The average SUVmax of LPL and MZL was 11.6 ± 3.2 and 12.1 ± 5, whereas in DLBCL it was 4.8 ± 1.7.

### 3.7. MCL

[^68^Ga]Ga-Pentixafor PET/MRI was also superior to [^18^F]FDG PET/MRI in the evaluation of MCL. In 19 patients who underwent both scans [24], [^68^Ga]Ga-Pentixafor PET/MRI and [^18^F]FDG PET/MRI sensitivity was 100% and 75.2%, respectively (*p* < 0.001), while positive predictive values were similar (94% vs. 96%, respectively; *p* = 0.21). SUVmax, SUVmean, TBRblood and TBRliver were significantly higher on [^68^Ga]Ga-Pentixafor PET than [^18^F]FDG PET. However, despite these facts, according to the Lugano classification, the stage of disease did not change between the two tracers for each patient. MCL is the lymphoma variant with the highest [^68^Ga]Ga-Pentixafor uptake, exceeding MZL, CLL, ALL and B-cell lymphoma. Only MM and adrenocortical carcinoma seem to have higher radiopharmaceutical uptake than MCL [27].

### 3.8. Other Lymphomas

Only two articles [16,21] about [^68^Ga]Pentixafor PET/MRI in CLL were available. In the first [16], thirteen CLL patients underwent [^68^Ga]Ga-Pentixafor PET/MRI and were compared with 20 controls (10 MALT lymphoma and 10 pancreatic adenocarcinomas). SUVmax and SUVmean of bone marrow (measured in the pelvis and in the lumbar vertebra L4) were significantly higher in CLL than pancreatic adenocarcinoma and MALT lymphoma. Instead, these semiquantitative parameters measured on the spleen or on the lesion with the highest uptake were not significantly different between these three oncological diseases. Furthermore, [^68^Ga]Ga-Pentixafor uptake in the bone marrow was associated with routine serum parameters (leukocyte count, lymphocyte percentage, lactate dehydrogenase, β2-microglobulin, and C-reactive protein) nor with apparent diffusion coefficient. This finding underlines the possibility that [^68^Ga]Ga-Pentixafor PET could have a role as an independent parameter for the detection, characterization, and treatment response assessment in CLL.

In the second study [21], the role of [^68^Ga]Ga-Pentixafor PET/MRI after Ibrutinib was studied: after less than one month of treatment, PET showed a reduction of uptake expressed a SUVmean in the bone marrow and lymph nodes, and an increased uptake in the spleen. After two to four months of therapy, PET demonstrated a decreased of uptake in the bone marrow and in the lymph nodes, while an increase in the spleen. These results are corresponding to the expression of CXCR4+ CLL cells that decreased in the bone marrow and nodes during therapy and accumulate in the splenic cavernous system.

Regarding T-cell lymphoma, DLBCL, and FL some preliminary results about the usefulness of [^68^Ga]Pentixafor PET were published but on a low sample of patients [19,23,27].

## 4. Discussion

Lymphomas are lymphoproliferative diseases with a high CXCR4 expression and are consequently suitable for evaluation with a CXCR4-targeted imaging technique. In vitro studies and xenograft models demonstrated the excellent affinity for the CXCR4 receptor and the direct correlation with CXCR4 receptor expression of [^68^Ga]Ga-Pentixafor PET [31,32,33]. The first human study [13] showed that [^68^Ga]Ga-Pentixafor PET was a highly selective and specific method for the in vivo quantification of CXCR4 expression and thus it can be of particular value for the pre-therapeutic confirmation of CXCR4 expression density prior to novel CXCR4 targeted therapies [34]. In this study, the authors observed excellent target to non-target ratios and derived the first dosimetry data, and corresponding analogs suitable for labeling with therapeutic β- or α-emitting radionuclides. Nowadays, [^18^F]FDG PET is considered the best noninvasive imaging tool for the staging, restaging, and treatment response evaluation of [^18^F]FDG-avid lymphoma, which are conventionally considered Hodgkin lymphoma (HL), FL, and DLBCL [35,36]. Instead, controversial results are available about [^18^F]FDG PET in other lymphoma variants, despite recent reports suggesting a good [^18^F]FDG-avidity of MCL [37], CNSL [38], and some subgroups of MZL [39]. Instead, other sub-types, such as WM/LPL and CLL, are defined as low-[^18^F]FDG avid lymphomas and consequently [^18^F]FDG PET/CT is not routinely recommended. For these lymphomas, CT is considered the gold standard imaging tool, but it is limited in evaluating metabolic/functional activity. Another potential field of application for [^18^F]FDG PET is radiotherapy planning and delivery.

For these reasons, a new radiotracer such as [^68^Ga]Ga-Pentixafor could help to study all lymphomas and in particular low-[^18^F]FDG avid lymphomas. In our review, we demonstrated that in almost all cases, the different lymphoma variants presented an increased [^68^Ga]Ga-Pentixafor uptake, and this finding was evident even for low-[^18^F]FDG-avid lymphomas. MCL and MZL (including MALT) seem to be lymphomas with higher [^68^Ga]Ga-Pentixafor uptake with high values of SUV and SUV ratios, but also CLL, CNSL and WM/LPL had increased avidity for this radiopharmaceutical. In comparison, [^68^Ga]Ga-Pentixafor PET demonstrated to have better performance than [^18^F]FDG PET/CT. These data are confirmed especially for lymphoma variants with low-moderate [^18^F]FDG uptake, such as LPL [17,19,26], CNSL [28], and MALT [22]. Additionally, for MCL [23] where [^18^F]FDG usually shows good accuracy, [^68^Ga]Pentixafor PET was superior. Aside from the detection rate/visual analysis, semiquantitative evaluation is also more efficient with [^68^Ga]Ga-Pentixafor than [^18^F]FDG, with average SUV and SUV ratios (with liver and blood pool as references) values significantly higher on [^68^Ga]Ga-Pentixafor PET due to a better lesion to background contrast. Despite SUV values, other types of metabolic parameters, such as metabolic tumor volumes have been studied only marginally [25] without deriving specifying cut-off values to stratify the risk or to make a differential diagnosis.

The same evidence may be carried out for texture analysis features, which has not yet been investigated for this new radiotracer.

A clear example of this advantage is in the evaluation of CNSL where the contrast between the lesion and the surrounding brain parenchyma is optimal with [^68^Ga]Ga-Pentixafor. Physiologically, the brain has increased [^18^F]FDG uptake which may reduce the evaluation of this organ in the search of hypermetabolic lesions, whereas this problem is not present for [^68^Ga]Ga-Pentixafor due to the very low/absent uptake in the normal brain. Despite the evidence, it seems to be premature to suggest a replacement of [^18^F]FDG PET with [^68^Ga]Pentixafor PET. Therefore, this hypothesis is worthy of further studies in a larger population. No data about HL and [^68^Ga]Ga-Pentixafor PET are present in the literature. Furthermore, for FL and DLBCL few cases were studied. Thus, it is not possible to express an opinion about the role of [^68^Ga]Ga-Pentixafor PET in all lymphoma histotypes. Another potential advantage of [^68^Ga]Ga-Pentixafor is the possibility to switch from the diagnostic to the therapeutic field with a different isotope profiting from the theranostic nature of this tracer. Until now, the only radionuclide therapy approved for relapsed low-grade or follicular B cell NHL is Yttrium-90 ibritumomab tiuxetan with preliminary positive results that are not very strong [40]. In this scenario, a new safety and efficient radionuclide therapy could have an impact. Regarding the evaluation of treatment response, which is fundamental for lymphoma and affects the following management, some preliminary evidence is described about [^68^Ga]Ga-Pentixafor PET. In CNSL, Starzer et al. [25] showed a perfect agreement between MRI and [^68^Ga]Ga-Pentixafor PET/MRI in the evaluation of residual disease after chemotherapy with a high dose of methotrexate. Also, Pan et al. [26] demonstrated optimal accuracy of [^68^Ga]Ga-Pentixafor PET in LPL patients after different chemotherapy protocols. Finally, Mayerhoefer et al. [29] evaluated the usefulness of [^68^Ga]GA-Pentixafor PET/MRI in gastric MALT lymphoma after Helicobacter Pylori eradication and derived excellent results with pooled accuracy, sensitivity, specificity, and positive and negative predictive values for the detection of residual gastric disease of 97%, 95%, 100%, 100%, and 92.9%. Until now, only one study about the prognostic role of [^68^Ga]Ga-Pentixafor PET is available [20] based upon 11 CNSL. This uncharted field could be a future research target with the aim of personalized evidence-based medicine. There are, however, challenges with dynamic and variable CXCR4 expression levels, indicating that further investigation of the receptor biology is required to fully understand the prognostic value and therapy response data. Small molecule alternatives to the peptidic agent, radiolabeled with either copper-64 or gallium-68, are undergoing preclinical evaluation and are likely to have a future clinical impact [41,42,43]. The scanner used does not seem to affect the diagnostic performance of [^68^Ga]Ga-Pentixafor for the study of lymphoma. In our review, both PET/CT and PET/MRI devices are utilized with similar findings. Only in one study [20] were these two techniques considered together without separate analysis. The advantages of a PET/MRI compared to PET/CT scanner are the low radiation exposure for patients and staff, excellent soft tissue contrast (helpful in several diseases), and simultaneous multi-modality imaging with a combination of anatomic and quantitative data.

The disadvantages are the less availability, the higher cost, lack of standardization due to the huge variations in MR protocols, longer acquisition time, the need of radiologists in loco, and the absence of shared metabolic criteria for the evaluation of examinations.

The increasing introduction into clinical practice of PET/CT scanners with silicon photomultiplier (SiPM) technology or new algorithms will likely lead to new advances in the field of functional imaging, but studies are desirable in this direction.

### Limitations of the Studies

Several limitations affect the quality of this systematic review such as the absence of multicentric studies, the low number of patients included, and the heterogeneity among the analyzed papers (for example for the scanner or for the lymphoma variants). This heterogeneity arises from variety in characteristics of the patients and lymphomas included and methodological aspects.

## 5. Conclusions

In spite of the heterogeneity of the studies and the wide variability in the sample analyzed, with this systematic review we can affirm that [^68^Ga]Ga-Pentixafor PET/CT or PET/MRI has a high detection rate and diagnostic performances for the evaluation of several lymphomas, especially MZL, CNSL, and LPL. Preliminary and similar evidences are available also for MCL and CLL. Moreover, in the evaluation of treatment response of some lymphomas, [^68^Ga]Ga-Pentixafor PET also seems to be a valid tool. However, more studies are needed to obtain definitive conclusions about the role of [^68^Ga]Ga-Pentixafor PET in lymphoma, in particular in comparison with [^18^F]FDG PET.

## Figures and Tables

**Table 1:** Main features of papers selected.

First Author	Year	Country	Study Design	N° Patients	M:F	Age Mean (Range)	Lymphoma Variants
Mayerhoefer, M.E. et al. [16]	2018	Austria	Prospective	13	7:6	65.6 (45–82)	13 CLL
Luo, Y. et al. [17]	2019	China	Prospective	17	11:6	62.5 (48–87)	17 WM/LPL
Haug, A.R. et al. [18]	2019	Austria	Prospective	36	17:19	62 (35–87)	36 MALT
Pan, Q. et al. [19]	2020	China	Retrospective	27	19:8	57.2 (15–76)	8 LPL; 4 MZL; 3 DLBCL; 2 FL; 6 T-cell; 1 MCL; 3 unclassified indolent B cell
Herhaus, P. et al. [20]	2020	Germany	Retrospective	11	8:3	64.1 (50–80)	11 CNSL
Mayerhoefer, M.E. et al. [21]	2020	Austria	Prospective	9	na	na	9 CLL
Duell, J. et al. [22]	2021	Germany	Retrospective	22	7:15	65 (50–80)	22 MZL (15 MALT)
Kuyumcu, S. et al. [23]	2021	Turkey	Retrospective	11	7:4	56.8 (22–80)	3 MCL; 1 MALT; 1 DLBCL; 2 CLL; 4 T cell
Mayerhoefer, M.E. et al. [24]	2021	Austria	Prospective	22	11:11	70 (52–82)	22 MCL
Starzer, A.M. et al. [25]	2021	Austria	Prospective	7	3:4	54.8 (30–79)	7 CNSL
Pan, Q. et al. [26]	2021	China	Prospective	15	12:3	60.9 (48–76)	15 WM/LPL
Buck, A.K. et al. [27]	2022	Germany & Austria	Retrospective	690 (220 lymphoma)	na	na	20 MCL; 187 MZL; 10 B-cell lymphoma; 3 T-cell lymphoma
Chen, Z. et al. [28]	2022	China	Retrospective	36	18:8	56.7 (18–77)	36 CNSL
Mayerhoefer, M.E. et al. [29]	2022	Austria	Prospective	26	14:12	64.1 (40–80)	26 gastric MALT

M: male; F: female; na: not available; DLBCL: diffuse large B cell lymphoma; CLL: Chronic Lymphocytic Leukemia; WM: Waldenstrom Macroglobulinemia; LPL: lymphoplasmacytic lymphoma; MALT: mucosa associate lymphatic tissue; MZL: marginal zone lymphoma; MCL: mantle cell lymphoma; FL: follicular lymphoma; CNSL: central nervous system lymphoma.

**Table 2:** Main technical aspects of studies included.

First Author	Device	Radiotracer Mean Injected Dose MBq	Uptake Time Min	Image Analysis	Semiquantitative Parameters
Mayerhoefer, M.E. et al. [16]	PET/MRI	150	60	Visual and semiquantitative	SUVmax; SUVmean
Luo, Y. et al. [17]	PET/CT	84.6	47.8	Visual and semiquantitative	SUVmax
Haug, A.R. et al. [18]	PET/MRI	172	60	Visual and semiquantitative	SUVmax; SUVmean; SUVpeak
Pan, Q. et al. [19]	PET/CT	2.8/Kg	56	Visual and semiquantitative	SUVmax; TBRblood; TBRliver
Herhaus, P. et al. [20]	PET/CT & PET/MRI	1–2.9/Kg	na	Visual and semiquantitative	SUVmax; TBR
Mayerhoefer, M.E. et al. [21]	PET/MRI	150	60	Visual and semiquantitative	SUVmax, SUVmean, PTV
Duell, J. et al. [22]	PET/CT	117	60	Visual and semiquantitative	SUVmax
Kuyumcu, S. et al. [23]	PET/CT	185	60	Visual and semiquantitative	SUVmax
Mayerhoefer, M.E. et al. [24]	PET/MRI	150	60	Visual and semiquantitative	SUVmax; SUVmean; TBRblood; TBRliver
Starzer, A.M. et al. [25]	PET/MRI	150	60	Visual and semiquantitative	SUVmax; SUVmean; PTV
Pan, Q. et al. [26]	PET/CT	85.1	46	Visual and semiquantitative	SUVmax
Buck, A.K. et al. [27]	PET/CT	134	60	Visual and semiquantitative	SUVmax; SUVmean, SUVpeak; TBR
Chen Z [28]	PET/CT	107	60	Visual and semiquantitative	SUVmax; T/N
Mayerhoefer ME [29]	PET/MRI	150	60	Visual and semiquantitative	SUVmax; SUVmean; TBRblood; TBRliver

MBq: megabecquerel; SUV: standardized uptake value; na: not available; TBR: tumor-to-background ratio; PTV: PET tumor volume; T/N: tumor to normal brain.
